# Reduced sensitivity of the SARS-CoV-2 Lambda variant to monoclonal antibodies and neutralizing antibodies induced by infection and vaccination

**DOI:** 10.1080/22221751.2021.2008775

**Published:** 2021-12-21

**Authors:** Meiyu Wang, Li Zhang, Qianqian Li, Bo Wang, Ziteng Liang, Yeqing Sun, Jianhui Nie, Jiajing Wu, Xiaodong Su, Xiaowang Qu, Yuhua Li, Youchun Wang, Weijin Huang

**Affiliations:** a Division of HIV/AIDS and Sex-transmitted Virus Vaccines, Institute for Biological Product Control, National Institutes for Food and Drug Control (NIFDC), Beijing, People’s Republic of China; b Graduate School of Peking Union Medical College, Beijing, People’s Republic of China; c Jiangsu Recbio Technology Co., Ltd., Taizhou, People’s Republic of China; d Beijing Advanced Innovation Center for Genomics (ICG) & Biomedical Pioneering Innovation Center (BIOPIC), Peking University; State Key Laboratory of Protein and Plant Gene Research, School of Life Sciences, Peking University, Beijing, People’s Republic of China.; e Translational Medicine Institute, The First People’s Hospital of Chenzhou, University of South China, Chenzhou, People’s Republic of China; f Department of Arboviral Vaccine, National Institutes for Food and Drug Control, Beijing, People’s Republic of China

**Keywords:** SARS-CoV-2, Lambda variant, C.37, neutralization, vaccines, convalescent serum, monoclonal antibodies

## Abstract

Severe acute respiratory syndrome coronavirus 2 variants have continued to emerge in diverse geographic locations with a temporal distribution. The Lambda variant containing multiple mutations in the spike protein, has thus far appeared mainly in South America. The variant harbours two mutations in the receptor binding domain, L452Q and F490S, which may change its infectivity and antigenicity to neutralizing antibodies. In this study, we constructed 10 pseudoviruses to study the Lambda variant and each individual amino acid mutation's effect on viral function, and used eight cell lines to study variant infectivity. In total, 12 monoclonal antibodies, 14 convalescent sera, and 23 immunized sera induced by mRNA vaccines, inactivated vaccine, and adenovirus type 5 vector vaccine were used to study the antigenicity of the Lambda variant. We found that compared with the D614G reference strain, Lambda demonstrated enhanced infectivity of Calu-3 and LLC-MK2 cells by 3.3-fold and 1.6-fold, respectively. Notably, the sensitivity of the Lambda variant to 5 of 12 neutralizing monoclonal antibodies, 9G11, AM180, R126, X593, and AbG3, was substantially diminished. Furthermore, convalescent- and vaccine-immunized sera showed on average 1.3–2.5-fold lower neutralizing titres against the Lambda variant. Single mutation analysis revealed that this reduction in neutralization was caused by L452Q and F490S mutations. Collectively, the reduced neutralization ability of the Lambda variant suggests that the efficacy of monoclonal antibodies and vaccines may be compromised during the current pandemic.

## Introduction

The coronavirus disease 2019 (COVID-19) pandemic, caused by severe acute respiratory syndrome coronavirus 2 (SARS-CoV-2), created an unprecedented global public health emergency within just a few months of its first report in December 2019 [1]. As of 22 September 2021, there have been more than 229 million confirmed cases of COVID-19 worldwide, including over 4.7 million COVID-19 related deaths [2]. Diverse therapies currently under investigation for COVID-19 include antiviral therapies (remdesivir, chloroquine) [3,4], anti-infectious therapies and anti-proinflammatory cytokines therapies (non-steroidal anti-inflammatory drugs) [5], but these drugs have limited therapeutic effect. Meanwhile, passive immunotherapies, such as convalescent plasma treatment are also in the clinical use, but are subject to the limitations of the long infusion time and high serum titre requirements [6]. Monoclonal antibodies (mAbs) that target the spike (S) protein, such as REGEN-COV and Sotrovimab, have a clinical benefit and have received emergency use authorization [7–9]. Although the above therapeutic strategies are important, the most effective way to control and end the COVID-19 pandemic is through safe and effective vaccination [10]. As of 19 September 2021, over 5.7 billion vaccine doses had been administered [2].

SARS-CoV-2 continues to evolve and has generated many variants. The World Health Organization (WHO) classifies variant viruses as variants of concern (VOC) and variants of interest (VOI). Currently, the WHO has recommended using letters of the Greek alphabet to rename variants, thus making naming simpler to understand and avoiding stigmatizing labels. The Alpha (B.1.1.7), Beta (B.1.351), Gamma (P.1), and Delta (B.1.617.2) variants are classified as VOC. The Eta (B.1.525), Iota (B.1.526), Kappa (B.1.617.1), Lambda (C.37), and Mu (B.1.621) variants have been classified as VOI [11]. The S protein, located on the surface of viral particles, is the main antigenic target of protective immune responses [12,13], with more than 90% of neutralizing antibodies in COVID-19 patients targeting the receptor binding domain (RBD) [14]. While these variants differ from one another to varying degrees, some S protein mutations are noticeably common to all variants. Specifically, D614G is the backbone of almost all variants, while N501Y is found in the Alpha, Beta and Gamma variants. Moreover, E484 mutations are commonplace in the Beta, Gamma, Eta, Iota and Kappa variants, while L452 mutations are found in the Delta, Kappa and Lambda variants. Of the Beta variants, the AY.1 and AY.2 lineages contain a K417N mutation, and the Gamma variant contains a K417 T mutation. These mutations can alter the transmissibility [15], disease severity [16] and antigenicity characteristics of the virus [17,18], thus posing a significant threat to the effectiveness of therapies and vaccine-induced immune protection [19–24].

The SARS-CoV-2 Lambda variant, first identified in Peru, is currently increasing in prevalence in the neighbouring countries of Chile, Argentina, and Ecuador, and has been detected in 42 countries [25]. In addition to D614G, the Lambda variant contains seven mutation sites in the S protein, including G75 V, T76I, Del246-252, D253N, L452Q, F490S, and T859N. Del246-252 is characterized as a unique deletion in the N terminal domain. L452Q and F490S are mutationsin antigenic sites recognized by antibodies. The L452R mutation found in the Delta, Kappa, and Epsilon variants has been reported to enhance viral infectivity and fusogenicity while promoting viral replication [26,27]. Meanwhile, the F490L mutation is resistant to some neutralizing antibodies [28], and the F490S mutation may affect the efficiency of existing vaccines [29]. However, it currently remains unknown whether the Lambda variant and its mutations evade antibody-mediated neutralization.

To study the Lambda variant and the effect of each individual amino acid mutation on viral function, we constructed a series of pseudoviruses. Four cell lines, Huh7, Calu-3, Vero and LLC-MK2, were used to detect infectious changes. Cell lines overexpressing ACE2 and furin, TMPRSS2, or cathepsin L (CatL) proteins were used to study the effects of these three enzymes on viral infection. Finally, mAbs and serum samples obtained from infected and vaccinated individuals were used to analyse changes in neutralization properties. This article only analyses neutralizing antibodies and does not detect T-cell responses or non-neutralizing antibodies.

## Materials and methods

### Plasmids

The mammalian codon-optimized S protein nucleotide sequence (GenBank: MN908947) was incorporated into the eukaryotic expression vector pcDNA3.1 using restriction endonucleases *Bam*HI and *Xho*I to obtain plasmid pcDNA3.1-Spike WT. The Lambda variant and individual amino acid mutation plasmids were then constructed using a point mutation method based on the pcDNA3.1-Spike WT expression plasmid, as previously described [28]. Briefly, the point mutation programme was based on the circular PCR method, and *Dpn*I (NEB, Ipswich, MA, USA) was used to digest template plasmids. The primers used to construct the G75 V, T76I, G75V + T76I, Del246-252+D253N, L452Q, F490S, L452Q + F490S, T859N, and Lambda variant plasmids are listed in Table 1.

### Cells

Four cell lines were used in this study: Huh-7 (Japanese Collection of Research Bioresources [JCRB], Osaka, Japan, JCRB0403), Vero (American Type Culture Collection [ATCC], Manassas, VA, USA; CCL-81), LLC-MK2 (ATCC; CCL-7) and Calu-3 (ATCC; HTB-55). Four stably overexpressing cell lines were used in this experiment. The cell line 293T-hACE2 comprised 293 T (ATCC; CRL-3216) cells stably expressing human ACE2. 293T-hACE2-Furin, 293T-hACE2-TMPRSS2 and 293T-hACE2-Cat L all stably overexpressed human ACE2 with additional overexpression of furin, TMPRSS2, and Cat L, respectively. All cells were cultured in Dulbecco's modified Eagle's medium (DMEM, high glucose; Hyclone, Logan, UT, USA) supplemented with 100 U/mL of penicillin–streptomycin solution (Gibco, Grand Island, NY, USA), and 10% foetal bovine serum (FBS; Pansera ES, PAN-Biotech, Aidenbach, Germany) at 37°C in a 5% CO_2_ environment. The cells were passaged every 2–4 days using trypsin-EDTA (0.25%; Gibco).

### Monoclonal antibodies

Twelve anti-SARS-CoV-2 S mAbs were used in this study. The mAb sources were mAbs AM180, A001 and M128 from Acro Biosystems Co. (Beijing, China); mAbs 4E5, 9A8 and 9G11 from Dr. Yuelei Shen of Beijing Biocytogen Inc. (Beijing, China); mAb AbG3 from Dr. Zhiqiang He of Fapon Biotech Inc. (Shenzhen, China); mAb MWF from Mabwell Bioscience Co. (Shanghai, China); mAbs X593 and X604 from Prof. Sunney Xie of Peking University; mAb CB6 from Prof. Jinghua Yan of the Institute of Microbiology, Chinese Academy of Sciences; and mAb R126 from Prof. Yongjun Guan.

### Sera from vaccinated participants

Nine serum samples were collected from individuals who had been immunized with the inactivated SARS-CoV-2 vaccine (KCONVAC, Vero cell; Shenzhen Kangtai Biological Products Co.; Shenzhen, China; Chinese Clinical Trial Registry: ChiCTR2000038804). Samples were collected 14 days after completion of standard immunization procedures (three doses at 0, 28 and 58 days; 5 µg/dose). The inactivated vaccine study protocol was approved by the Ethics Committee of the Jiangsu Provincial Centre for Disease Control and Prevention. On May 8, 2021, National Medical Products Administration of China announced the conditional approval of the KCONVAC vaccine for marketing and use [30].

Five serum samples were collected from individuals who had been immunized with the Recombinant adenovirus COVID-19 vaccine (Convidecia, Adenovirus Type 5 Vector, CanSino Biologics Inc. ChiCTR2000031781). Samples were collected 28 days after the immunization procedure (one dose at 0 days; 300 µl/dose). The study protocol of Convidecia was approved by the Ethics Committee of Jiangsu Provincial Centre for Disease Control and Prevention. On February 25, 2021, National Medical Products Administration of China announced the conditional approval of the registration application for Convidecia vaccine [31–33].

Stemirna Company (Shanghai, China) provided four serum samples from individuals who had been immunized with a novel coronavirus mRNA vaccine (LPP/mRNA-Spike) [34]. Samples were collected 14 days after completion of immunization procedures (two doses at 0 and 21 days; 25 µg/dose).

Suzhou Abogen Biosciences (Suzhou, China) provided five serum samples from individuals who had been immunized with a thermostable mRNA vaccine against COVID-19 (ARCoV-RBD) [35]. Samples were collected 14 days after completion of immunization procedures (two doses at 0 and 28 days; 15 µg/dose).

### Convalescent sera

Fourteen convalescence serum samples were collected between March 2020 and October 2020 and provided by Prof. Xiaowang Qu of the University of South China (Hengyang, China). Written informed consent was obtained from all patients for serum collection.

### SARS-CoV-2 pseudoviruses

Pseudoviruses were prepared as previously described [36]. Briefly, pseudoviruses were generated by transfection with pcDNA3.1-Spike WT and concurrent infection with G*ΔG-VSV (Kerafast, Boston, MA, USA). The cell supernatant containing SARS-CoV-2 pseudoviruses was harvested 24 and 48 h later, then stored at −80°C.

### Infection and neutralization assays

As we described previously paper [37], for infection assays, similar copy numbers of different SARS-CoV-2 variant pseudoviruses were mixed with the indicated cells. Specifically, SARS-CoV-2 variant pseudoviruses were quantified via RT–PCR using a vesicular stomatitis virus phosphoprotein plasmid as an internal control. Pseudoviruses of the Lambda variant and each individual mutation were diluted to the same number of copies before use in experiments.

For neutralization assays, mAbs or serum samples were serially diluted and preincubated with pseudoviruses for 1 h at 37°C, then mixed with Huh 7 cells. The cells were then incubated at 37°C for 24 h, in a 5% CO_2_ environment. Chemiluminescence signals were collected by PerkinElmer Ensight using luciferase substrate (PerkinElmer, Waltham, MA, USA). Each experiment was repeated three or four times.

### Structural modelling

The spike protein was modelled based on the following Protein Data Bank coordinate sets: RBD-Ab5 for 9G11 and 7chh for X593. Mutant simulations were performed on the Mutabind2 web server. MutaBind2 calculates changes in binding affinity following single or multiple mutations and provides structural models of mutant complexes. MutaBind2 models use molecular mechanics force fields, statistical potentials, and a fast side chain optimization algorithm constructed via a random forest method. Protein–protein interactions were calculated in PDBePISA, and images demonstrating structures were generated in PyMOL.

### Statistical analysis

The 50% inhibitory dose (ID50) was calculated using the Reed-Muench method. Graphical representations and statistical analyses were generated using GraphPad Prism 8 (GraphPad, San Diego, CA, USA). Violin plots were used to display the distribution status and probability density of multiple data groups. One-way ANOVA and Holm–Sidak's multiple comparisons test were used to analyse differences relative to the D614G reference strain. *P*<0.05 was considered statistically significant. For all figures, the following notations are used: * *P*<0.05, ** *P*<0.01, *** *P*<0.001, and **** *P*<0.0001.

## Results

### Infectivity of the Lambda variant

**Figure 1.**
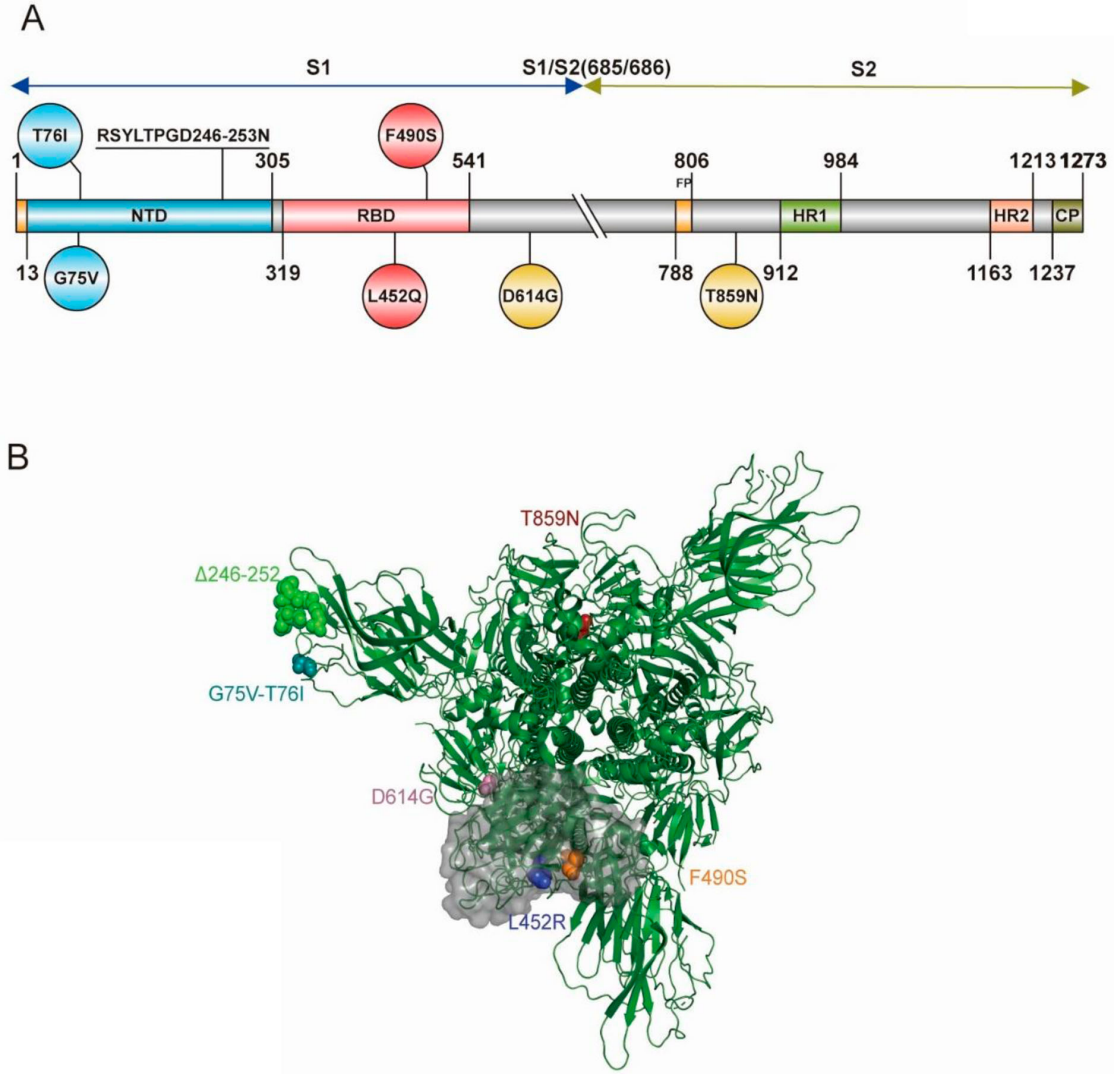
**Schematic illustration of the spike protein of the SARS-CoV-2 Lambda variant** (A) Domain structure of the Lambda variant, including amino acid changes. Circles indicate an amino acid mutation at that position; a mutation with seven amino acid deletions is indicated by underlined text. NTD, N-terminal domain; RBD, receptor-binding domain; FP, fusion peptide; HR1, heptad repeat 1; HR2, heptad repeat 2; CP, cytoplasmic domain. The S1/S2 cleavage site is indicated with a double slash at the 685/686 site of the Spike protein. (B) The 3D structure of the Lambda variant.

**Figure 2.**
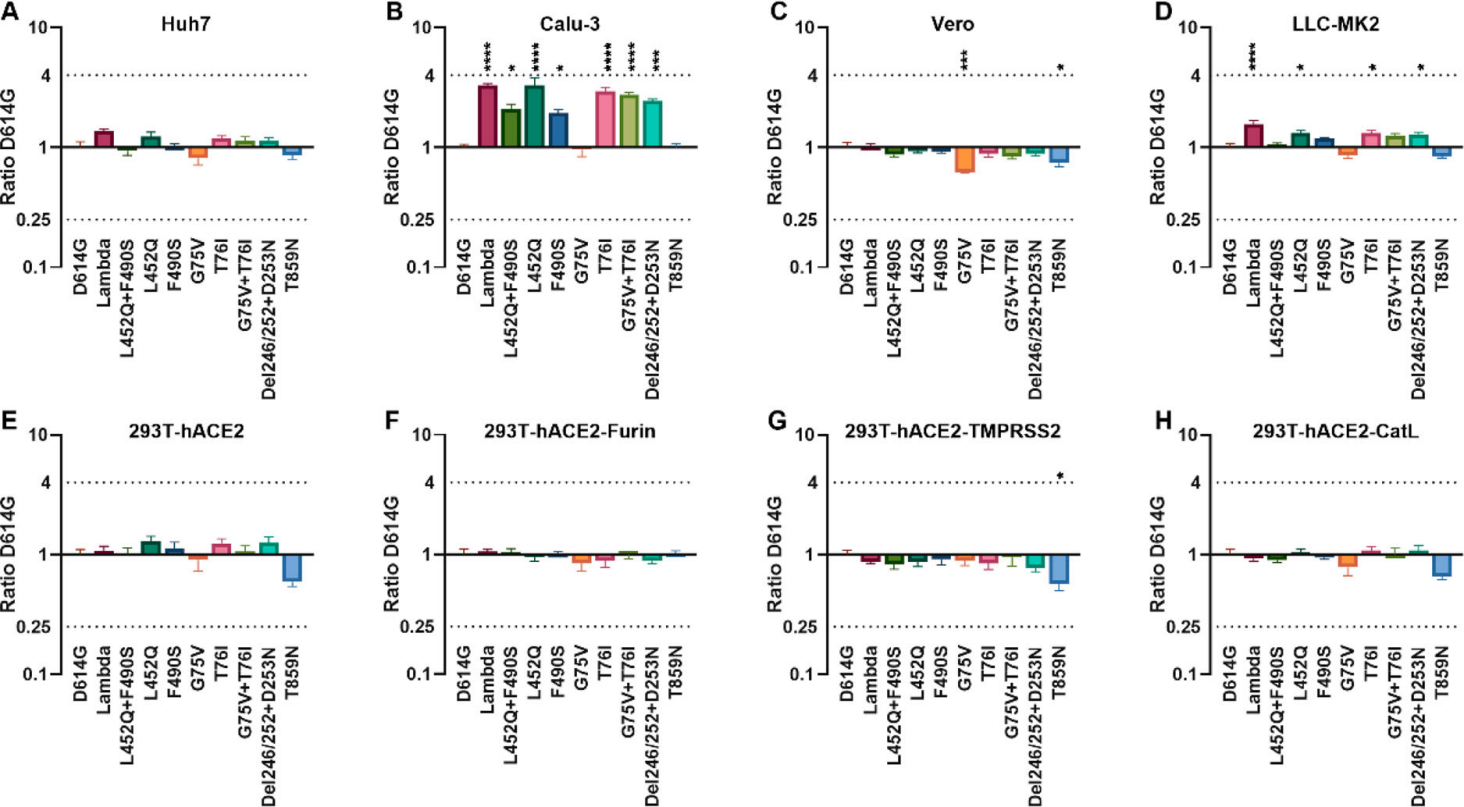
**Infectivity analysis of Lambda variant pseudoviruses** Infection assays with the 10 Lambda variant-related mutant pseudotyped viruses in Huh-7 (A), Calu-3 (B), Vero (C), LLC-MK2 (D), 293T-ACE2 (E), 293T-ACE2-Furin (F), 293T-ACE2-TMPRSS2 (G) and 293T-ACE2-CatL (H) cells. The relative infectivity of the mutant pseudoviruses is presented as the ratio of relative luciferase units (RLU) for mutant/RLU for the D614G variant. All results were obtained from three independent experiments (mean ± SEM). The dashed lines indicate the threshold of four-fold differences. One-way ANOVA and Dunnett's multiple comparisons test were employed to determine differences in infectivity between Lambda and D614G.

To study the Lambda variant and each individual amino acid mutation's effect on viral function, we constructed 10 pseudovirus strains using the pseudovirus technique, including the D614G mutation, G75 V, T76I, Del246/252+D253N (RSYLTPGD246-253N), L452Q, F490S, T859N, L452Q + F490S, G75V + T76I and Lambda variant based on the D614G background (Figure 1). To investigate whether the infectivity of different cell lines was altered after virus mutation, we used the human hepatocellular carcinoma cell line Huh7, human lung adenocarcinoma cell line Calu-3, African green monkey kidney cell line Vero, and rhesus monkey kidney cell line LLC-MK2 for pseudoviral infection experiments (Figure 2A–D). We found a 1.4-fold enhancement in infection by the Lambda variant in Huh7 cells compared with the D614G mutant strain, but this was not statistically significant. No significant differences in the infectivity of the pseudoviruses with single point mutations in Huh7 cells existed. However, the Lambda variant showed a 3.3-fold enhanced ability to infect Calu-3 cells (*P*<0.0001), which was statistically significant. Moreover, the infection of Calu-3 cells by L452Q, F490S, T76I and Del246/252+D253N pseudoviruses was enhanced 3.3-fold, 1.9-fold, 2.9-fold, and 2.4-fold, respectively. However, the Lambda variant showed no significant change in cell infection and was less infectious than G75 V and T859N pseudoviruses. Additionally, the Lambda variant demonstrated 1.6-fold enhanced infection of LLC-MK2 (*P*<0.0001) and showed significantly enhanced infectivity of L452Q, T76I and Del246/252+D253N pseudoviruses.

Subsequently, we investigated the role of three enzymes, furin, TMPRSS2, and cathepsin L, during viral infection. We transfected 293 T cells with the ACE2 receptor and superimposed these with overexpression of one of the three proteases, before infection with the 10 pseudoviruses described above (Figure 2E–H). No significant changes existed in the Lambda variant's ability to infect these four cell lines compared with the D614G pseudovirus. However, we found that infection of T859N pseudovirus on 293T-ACE2, 293T-ACE2-TMPRSS2, and 293T-ACE2-CatL were all reduced by 1.7-fold, 1.8-fold, and 1.5-fold, respectively.

### Neutralization of the Lambda variant by neutralizing mAbs

**Figure 3.**
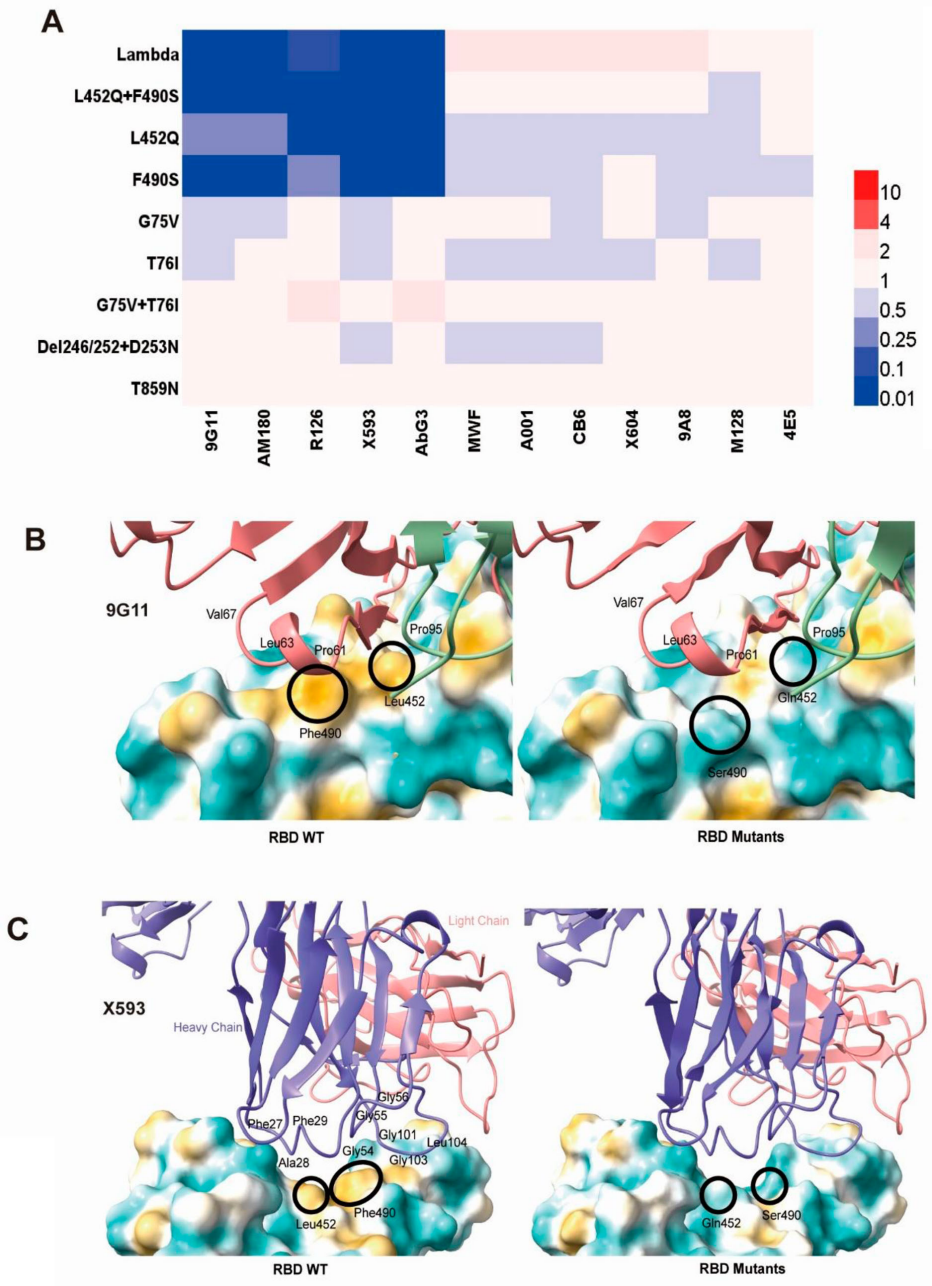
**Analyses of Lambda variant antigenicity using a panel of neutralizing mAbs** (A) Heatmap representation of neutralization reactions using 12 neutralizing mAbs against 10 Lambda-related mutant pseudoviruses. The ratio of ID50 value between Lambda and the D614G reference was calculated and analysed, followed by heatmap construction using HemI software [38]. Data represent three independent experiments. Red and blue boxes represent increased and decreased viral sensitivity to mAbs, respectively, as shown in the scale bar. A 4-fold difference was considered significant. Structural modelling of the L452Q and F490S mutations, based on RBD-9G11 for 9G11 (B), and RBD-X593 for X593 (C). The colour of the RBD ranges from yellow to white to blue indicating hydrophobic to hydrophilic, and the images were produced in UCSF ChimeraX.

To investigate differences in Lambda variant antigenicity compared with the SARS-CoV-2 reference strain, we first selected 12 neutralizing mAbs against the SARS-CoV-2 S protein and performed neutralization experiments on our 10 pseudoviruses (Figure 3A, Figure S1). We found that five mAbs showed decreased or absent neutralizing protection against the Lambda variant. Among them, mAb 9G11 showed a 41.7-fold reduction in neutralizing protection against Lambda variants, mainly caused by the F490S mutation, followed by L452Q. The neutralizing protection of mAb AM180 decreased more than 243-fold, an almost complete abrogation of protection, which was caused by the accumulated mutations of F490S and L452Q. Meanwhile, the neutralizing effects of mAbs R126, X593, and AbG3 decreased 7.7-fold, 129.2-fold, and 16.9-fold, respectively. The decreased neutralizing effect of these three mAbs resulted from a combination of L452Q and F490S mutations in the RBD region. In addition, the seven mAbs MWF, A001, CB6, X604, 9A8, M128, and 4E5 did not differ more than 4-fold in their neutralization of the Lambda variant compared with their neutralization of D614G, indicating that these seven mAbs provided good protection against the Lambda variant.

### Neutralization of the Lambda variant by convalescent sera

**Figure 4.**
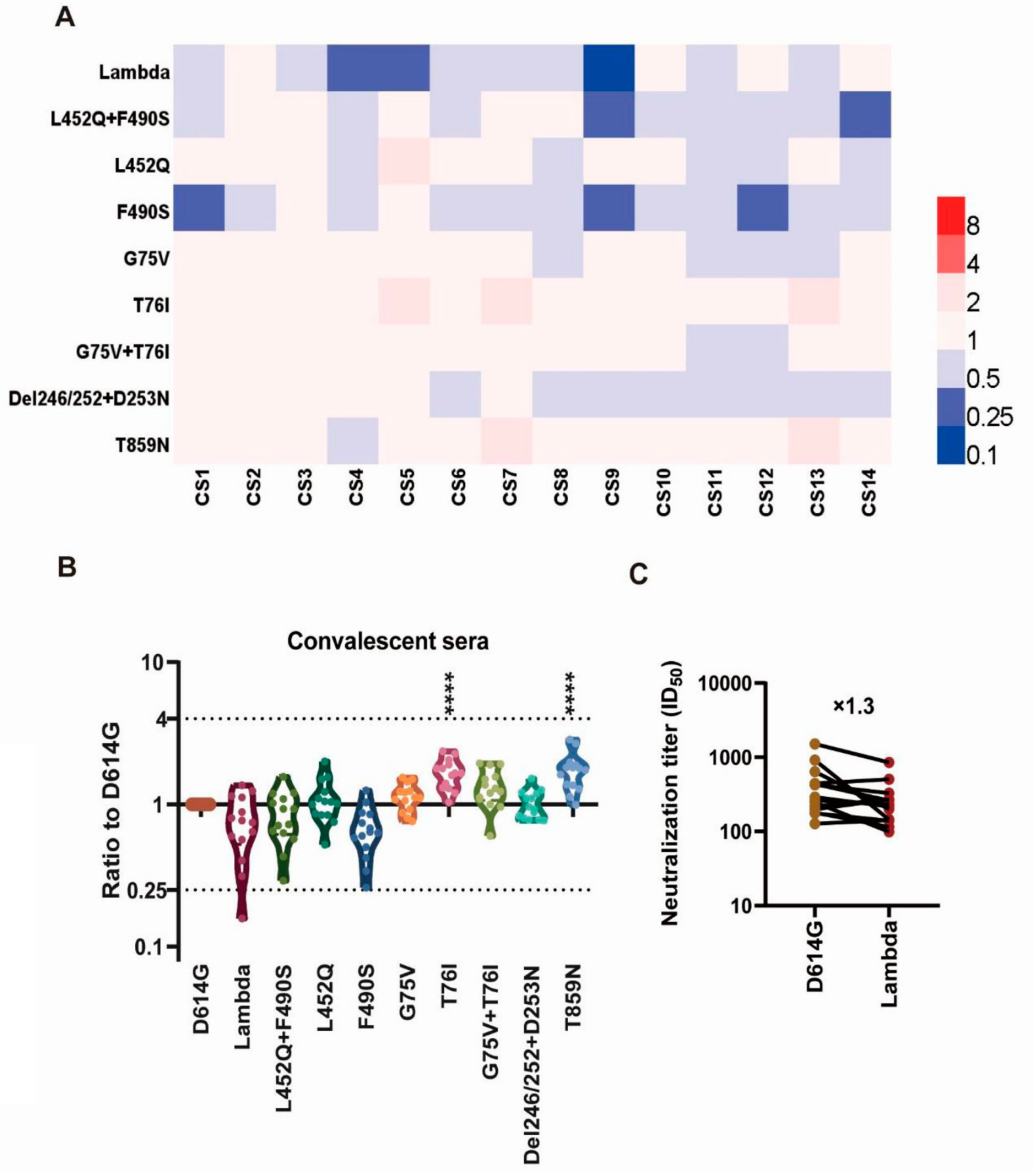
**Analysis of the antigenicity of the Lambda variant using a panel of convalescent sera** (A) Heatmap representation of the neutralization reactions using 14 convalescent sera (CS1-14) against 10 Lambda-related mutant pseudoviruses. The ratio of ID50 values (mean of three independent experiments) between the Lambda variant and D614G reference was calculated and analysed, followed by construction of a heatmap using HemI software. Red and blue boxes represent increased and decreased viral sensitivity to convalescent sera, respectively. (B) Violin plot of the summary and statistical analysis of changes in antigenicity of the Lambda variant. Each dot (mean of three independent experiments) represents one convalescent serum. One-way ANOVA and Holm-Sidak's multiple comparison tests were used to analyse the differences between groups. A *P* value less than 0.05 was considered statistically significant. * *P*<0.05, ** *P*<0.01, *** *P*<0.001, and **** *P*<0.0001. (C) Analysis of the ID50 values of neutralization reactions between the D614G reference and Lambda pseudoviruses to convalescent sera.

We further investigated the antigenic changes of the Lambda variant using convalescent sera from patients who had been infected with SARS-CoV-2. We selected 14 convalescent sera derived from different infected individuals and performed neutralization tests against the 10 pseudoviruses (Figure 4A). We found no significant differences in the neutralization of Lambda compared with D614G by convalescent sera, with only an approximately 1.3-fold reduction (Figure 4B, C). Moreover, the convalescent sera's neutralizing effect against F490S was reduced by 1.5-fold, while the neutralizing protection against L452Q was unchanged (Figure 4B). Furthermore, we found that T76I and T859N pseudoviruses were more easily neutralized by convalescent sera, both with 1.7-fold increased sensitivity (Figure 4B), which was statistically significant (*P*<0.0001).

### Protection from the Lambda variant by vaccine-immunized sera

**Figure 5.**
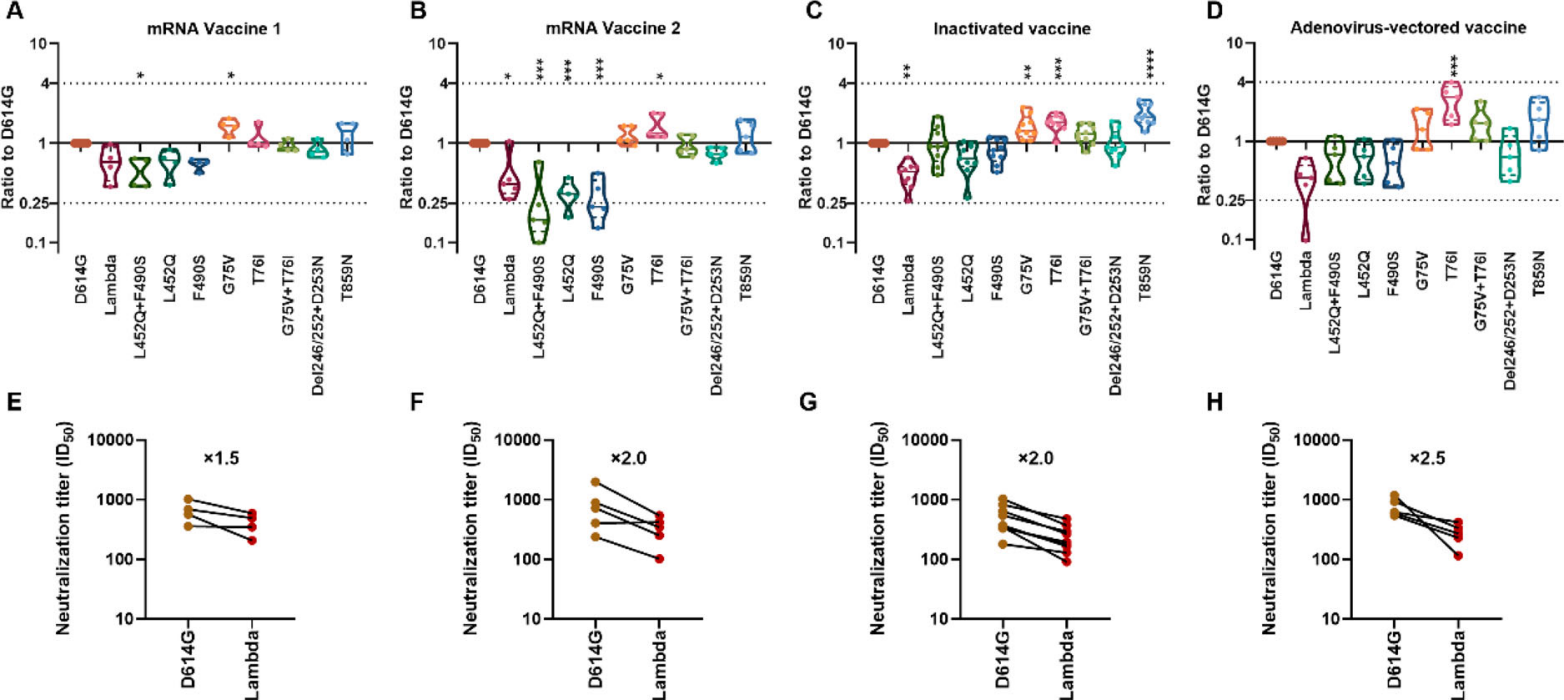
**Analysis of the antigenicity of the Lambda variant using a panel of sera after immunization with different vaccine types** Violin plot of the neutralization reactions against 10 Lambda-related mutant pseudoviruses using immune sera following immunization with different vaccine types (A, B, C, D). (A) mRNA vaccine 1: LPP/mRNA-Spike mRNA vaccine-immunized sera; (B) mRNA vaccine 2: ARCoV-RBD mRNA vaccine-immunized sera; (C) Inactivated vaccine: KCONVAC inactivated SARS-CoV-2 vaccine-immunized sera; (D) Adenovirus-vectored vaccine: Ad5 adenovirus-vectored vaccine-immunized sera. Analysis of the ID50 values of the neutralization reactions for vaccine-immunized sera between D614G and Lambda pseudoviruses (E, F, G, H). (E) mRNA vaccine 1: LPP/mRNA-Spike mRNA vaccine-immunized sera; (F) mRNA vaccine 2: ARCoV-RBD mRNA vaccine-immunized sera; (G) Inactivated vaccine: KCONVAC inactivated SARS-CoV-2 vaccine-immunized sera; (H)Adenovirus-vectored vaccine: Ad5 adenovirus-vectored vaccine-immunized sera. The data were the results from three replicates.

To study the protective effect of vaccine-immunized sera against the Lambda variant, 23 vaccine-immunized sera were collected, including nine mRNA vaccine-immunized sera (Stemirna Company, n=4; Suzhou Abogen Biosciences, n=5), nine inactivated vaccine-immunized sera, and five Ad5 adenovirus-vectored vaccine-immunized sera (Figure 5).

Stemirna Company's mRNA vaccine-immunized sera showed a 1.5-fold decrease in neutralization against the Lambda variant and 1.5-fold and 1.6-fold decreases against the RBD region L452Q and F490S mutant pseudoviruses, respectively (Figure 5A, E). Meanwhile, the neutralization effect against the L452Q + F490S combined amino acid mutant pseudovirus was decreased by 1.9-fold, which was statistically significant (*P*=0.044). Futhermore, the neutralization effects of these sera against the G75 V mutant strain were enhanced 1.5-fold, which was also statistically significant (*P*=0.038).

Suzhou Abogen Biosciences's mRNA vaccine-immunized sera showed a significantly decreased (2-fold, *P*=0.024) neutralization effect against the Lambda variant (Figure 5B, F). Similarly, the neutralization effects against the L452Q, F490S and L452Q + F490S pseudoviruses were decreased by 3.2-fold, 3.5-fold and 3.8-fold, respectively(*P*<0.001). Conversely, the neutralizing effect of the sera against the T76I mutant strain was enhanced 1.5-fold, which was statistically significant (*P*=0.021).

The nine inactivated vaccine-immunized sera showed a significant 2.0-fold decrease (*P*=0.007) in neutralization of the Lambda variant (Figure 5C, G). However, no significant changes occurred in neutralization of the RBD amino acid mutant strains L452Q and F490S, which decreased 1.4-fold and 1.2-fold, respectively. In contrast, G75 V, T76I, and T869N pseudoviruses appeared to be more easily neutralized, with 1.5-fold (*P*=0.01), 1.6-fold (*P*=0.0007), and 2.0-fold (*P*<0.0001) enhanced neutralization, respectively, which were all statistically significant differences.

The neutralizing effect of the five Ad5 adenovirus-vectored vaccine-immunized sera against the Lambda variant decreased by an average of 2.5-fold, but this was not statistically different from the effect observed against D614G because of the large differences between samples (Figure 5D, H). Meanwhile, no significant changes existed in the neutralization effect of vaccine-immunized sera against the L452Q, F490S and L452Q + F490S mutant strains, showing 1.5-fold, 1.6-fold, and 1.4-fold decreases, respectively. Furthermore, the neutralizing effects of these sera against the T76I mutant strain were enhanced 2.7-fold, which was statistically significant (*P*=0.0002).

## Discussion

The Lambda variant (C.37) was first discovered in Peru, then rapidly spread through South America, before reaching countries globally. It was defined as a VOI by the WHO on June 14, 2021, because the mutations in its viral genome were presumed to cause immune or therapeutic escape. As of September 9, 2021, other variants defined as VOI included B.1.525, B.1.526, B.1.617.1 and B.1.621. The reduced neutralizing activity of BNT162b2 vaccine-immunized sera against B.1.525, B.1.617.1, and B.1.621 has been reported, thus affecting the protective effects of the vaccine [21,39]. Although two studies on pseudoviruses have suggested that the neutralization activity of mAb and mRNA vaccine-induced sera against the Lambda variant may be decreased, the protective effects of mAbs, mRNA, inactivated, and adenoviral vector vaccines against the Lambda variant remain unclear. Therefore, we investigated the antigenic and infectivity changes of Lambda based on pseudovirus techniques.

In this study, we found that the infectivity of the Lambda variant was enhanced in Calu-3 cells, while no significant infection enhancement was observed in other cell lines. This phenomenon may be because Calu-3 cells, like airway epithelial cells, express TMPRSS2, required for SARS-CoV-2 entry, and a small amount of CatL [40,41]. Several studies have demonstrated that the virus behaves differently in Calu-3 cells than in other cell lines, such as Vero and VeroE6 [42–44]. However, Lambda had no effect on 293 T cells overexpressing TMPRSS2 or CatL proteases. In pseudovirus infection experiments, we found that the luminescence value of Calu-3 cells was much lower than that in protease overexpressing cell lines. Calu-3 cells are a directly isolated cell line with protease expression that is more comprehensive and closer to human cell expression levels. Conversely, cell lines that overexpress large numbers of proteases allow for much more efficient SARS-CoV-2 infection and expression than Calu-3 cells. The pseudovirus's high infectivity to protease overexpressing cells may have masked subtle differences in infectivity caused by Lambda. In addition, we found a 1.6-fold decrease in the infectivity of G75 V in Vero cells. Meanwhile, the infectivity of T859N was decreased 1.3-fold, 1.7-fold, 1.5-fold, and 1.8-fold in Vero, 293T-hACE2, 293T-hACE2-TMPRSS2, and 293T-hACE2-CatL cells, respectively, which is consistent with a previous report [45]. Furthermore, our findings showed no significant difference in the infection of ACE2 overexpressing cells by Lambda compared with the D614G variant. By contrast, two previous studies reported enhanced infectivity of Lambda on ACE2 overexpressing cell lines [45,46]. It is noteworthy that Lambda's enhanced infectivity was around 1.5-fold in these two articles. In other words, Lambda's change in infectivity was much less pronounced than that of D614G, which was about 10 times higher than the initial epidemic virus [28].

In our study of neutralizing antibodies, we found five mAbs that showed reduced protection against the Lambda variant. Among them, immune escape from mAbs 9G11 and AM180 by Lambda was caused by both L452Q and F490S mutations, with immune escape for F490S being stronger than for L452Q. Our previous study showed that both F490 and L452 are located at the binding site of mAb 9G11 to the RBD, and experimentally verified that F490L and L452R mutations can cause escape from mAb 9G11, which is consistent with the results presented here [47]. In addition, escape from mAb R126 was caused by both L452Q and F490S mutations, with L452Q exerting a greater effect than F490S. Furthermore, escape from mAbs X593 and AbG3 by the Lambda variant was caused by both L452Q and F490S mutations. Our previous study found that the L452R and F490L mutations can cause escape from mAb X593, consistent with the results of the L452Q and F490S mutations in the present study [28]. Taken together, our results suggest that the L452R and L452Q mutations, and the F490S and F490L mutations, have the same effect on immune escape from mAbs.

Previous studies showed that L452R increases viral infectivity and fusion, while also promoting viral replication [27]. Simultaneously, L452R can escape cellular immunity [27], while neutralizing protection against L452R by mAbs, convalescent sera, and vaccine-induced immune sera is significantly reduced [48,49]. Therefore, we hypothesize that the Lambda variant containing the L452Q mutation is highly variable in both infectivity and antigenicity, warranting close monitoring and attention. In addition, our previous studies revealed that escape of the L452R and E484 K mutants from mAbs frequently occurred in conjunction. In the present study, we found that the escape of L452Q and F490S mutants from mAbs also appeared concomitantly. It is likely that L452 and F490 are often located simultaneously within the recognition epitopes of certain mAbs against the S protein of SARS-CoV-2.

To further analyse the reason for the failure of a mAb to neutralize the test variant, we performed structural analysis of the mAb–RBD complex based on the reported structural information. L452 and F490 on the RBD do not bind directly to mAb 9G11 (RBD–Ab5 for 9G11), but are involved in binding primarily with the hydrophobic core that forms a cross-interface with hydrophobic properties [47]. When the mutation of L452Q with F490S occurs, it breaks the original hydrophobic interface. Although new hydrogen bonding occurs, the newly generated hydrogen bonds are not sufficient to counteract the effects of disruption of the hydrophobic interface, resulting in the escape of mAb 9G11 (Figure 3B). Similarly, mAb X593 binding to the RBD is dependent on salt bridges and hydrogen bonds (7chh for X593), and we found that L452 and F490 are not directly involved in the interaction and mainly form a cross-interface with the hydrophobic amino acids in the CDRH region of the mAb X593 hydrophobic core [50]. When the L452Q and F490S mutations occurred, the hydrophobic interface responsible for binding on RBD was broken, resulting in the escape of the Lambda variant from binding to mAb X593 (Figure 3C).

Several reports have proposed that those infected with SARS-CoV-2 can better resist emerging variants than vaccine-immunized populations [51–53]. Therefore, it is important to study the changes in convalescent sera to Lambda variant. There is only one published study on convalescent sera, which found a 3.3-fold decrease in neutralizing titres against the Lambda variant, a 4.4-fold decrease against L452Q, and a 2.5-fold decrease against F490S [46]. Our study also found a decrease in the neutralizing protection of convalescent sera against the Lambda variant; however, this was only a 1.3-fold decrease, which was not a statistically significant different. In addition, the previous study compared the difference in neutralizing protection of convalescent sera between the Beta and Lambda variants and found a 4.9-fold decrease in neutralizing protection against Beta and a stronger neutralization effect against Lambda [46]. In our previous study of the Beta variant, we found that the higher the sera neutralization titre, the more pronounced the decrease in neutralizing protection against Beta [37]. Thus, the smaller change in protection against the Lambda variant by convalescent sera in our study may be due to the lower neutralizing titres of the convalescent sera used. Neutralizing antibody levels are highly predictive of immune protection from symptomatic SARS-CoV-2 infection. Therefore, our results infer that individuals previously infected with SARS-CoV-2 in South America are still protected against emerging Lambda variant infections.

Vaccination efforts are currently being accelerated worldwide, but the constant mutation of SARS-CoV-2 poses a great challenge to the protective effect of any administered vaccine. Studying the correlation between laboratory results with sera after vaccine immunization and clinical protective effects is essential to explore the protective mechanisms of the COVID-19 vaccine. Recent studies have analysed the relationship between in vitro neutralization levels and observed protection against SARS-CoV-2 infection using data from seven current vaccines and convalescent cohorts. The data show that neutralizing antibody levels are highly predictive of immune protection from symptomatic SARS-CoV-2 infection [54]. Therefore, the results of our *in vitro* neutralization tests on vaccine-immunized sera to Lambda variant are informative for researchers, physicians, and public health workers. In this study, three types of vaccine-immunized sera from four manufacturers were used to investigate changes in the neutralization of the Lambda variant. Recently, two pseudovirus-related studies showed a 3.0-fold and 1.5-fold decrease in neutralization of Lambda by BNT162b2 vaccine-immunized sera and a 2.3-fold decrease in neutralization of Lambda by mRNA-1273 vaccine-immunized sera [45,46]. In another study, the titre of RBD-conjugated antibodies from mRNA-vaccinated donors to the Lambda variant decreased by 1.5–1.9-fold [55]. Our study found 1.5-fold and 2.0-fold decreases in neutralization of the Lambda variant by mRNA vaccine-immunized sera from Stemirna Company and Suzhou Abogen Biosciences, respectively, which is consistent with previous reports. However, in China, most vaccinated individuals have received inactivated vaccines. A study using the CoronaVac inactivated vaccine showed a 3.05-fold decrease in neutralizing protection against the Lambda variant by vaccine-induced immune sera compared with the wild-type 614D strain [56]. The 2.0-fold decrease in neutralizing protection against the Lambda variant by the KCONVAC inactivated vaccine-induced immune sera in the present study was also more consistent with the results of Acevedo and colleagues [42]. Evaluation of the Ad5 adenovirus-vectored vaccine showed that there was a 2.5-fold decrease in neutralization titre against the Lambda variant, which was the largest decrease among the vaccines involved in this study. In the study of convalescent sera and vaccine-immunized sera, we found only a 1.3-fold decrease in neutralizing activity of convalescent sera against Lambda, whereas vaccine-immunized sera showed a 1.5–2.5-fold decrease. The reason for this phenomenon may be the diversity of antibodies produced in naturally-infected individuals. The results also suggest that the naturally-infected population may be better able to resist infection with the Lambda variant than the vaccine-immunized population [51–53]. Although the number of vaccine types used in this study was large, the number of serum samples immunized with each vaccine was small, and the sample size needs to be increased for further research.

In conclusion, our data suggest that neutralizing protection by mAbs, convalescent sera and vaccine immunized sera is reduced against the Lambda variant. Our findings provide insight for the development of therapeutic antibodies and vaccines. Therefore, in vitro detection of changes in neutralization of mutant strains by mAbs should be performed when using mAbs clinically. In addition, although the neutralizing activity of vaccine-immunized sera was decreased, the antibody titre remained elevated. Therefore, the Lambda variant may not have a significant impact on the protective effect of current vaccines.

## Supplementary Material

Supplemental Material

Table_S2.xlsx

Table_S1.xlsx

Figure_S1.tif
