# Supplementary material for: Reduced sensitivity of the SARS-CoV-2 Lambda variant to monoclonal antibodies and neutralizing antibodies induced by infection and vaccination
Source: Emerg Microbes Infect. 2021 Dec 21;11(1):18–29. doi: 10.1080/22221751.2021.2008775 (PMC8725979; doi:10.1080/22221751.2021.2008775)
Supplement: Supplemental Material [file TEMI_A_2008775_SM8821.docx]

**Supplementary material**

**Figure S1. Analyses of Lambda variant antigenicity using a panel of neutralizing mAbs. Related to Figure 3.**

Histogram of the changes in antigenicity of the Lambda variant to the mAbs. The ratio of the ID50 values between Lambda and the D614G reference was calculated and analysed, followed by construction of a histogram using GraphPad software. The data (mean ± SEM) derived from three replicates. The y axis represents the ID50 ratio. The vertical dashed lines indicate the threshold of four-fold differences.

**Table S1. Primers for construction of the SARS-CoV-2 Lambda variant**

Forward primers for site-directed mutations are listed. Reverse primers are the reverse complement of the forward primers and are not listed in this table.

**Table S2. Information on the convalescent sera used in the neutralization assays**
