# Supplementary figures and images for: Reduced sensitivity of the SARS-CoV-2 Lambda variant to monoclonal antibodies and neutralizing antibodies induced by infection and vaccination
Source: Emerg Microbes Infect. 2021 Dec 21;11(1):18–29. doi: 10.1080/22221751.2021.2008775 (PMC8725979; doi:10.1080/22221751.2021.2008775)

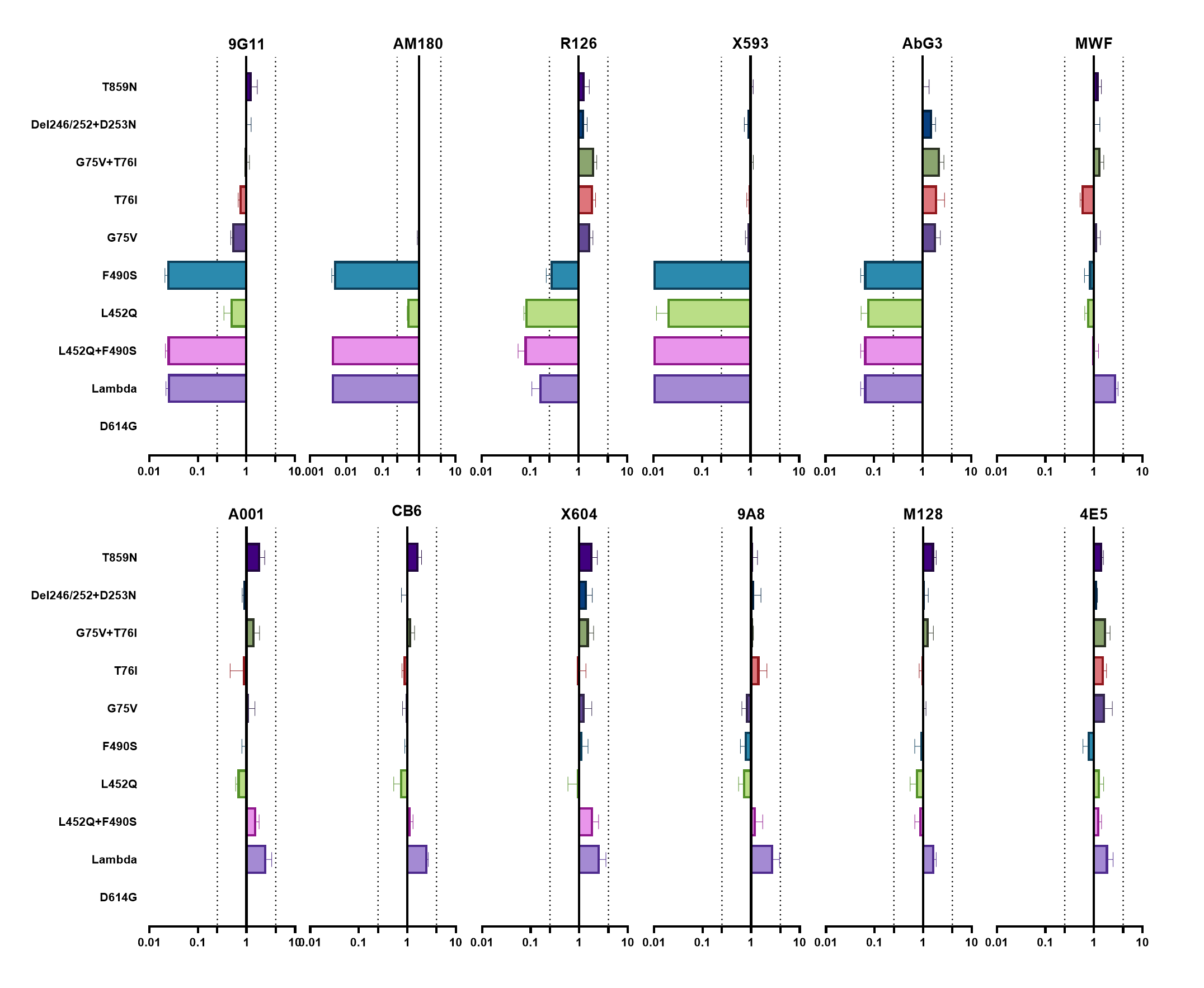

Supplement: Figure_S1.tif [file TEMI_A_2008775_SM3357.tif]
